# Incidence and clinical characteristics of ciguatera fish poisoning in Guadeloupe (French West Indies) between 2013 and 2016: a retrospective cases-series

**DOI:** 10.1038/s41598-018-21373-2

**Published:** 2018-02-15

**Authors:** Denis Boucaud-Maitre, Jean-Paul Vernoux, Stéphane Pelczar, Elise Daudens-Vaysse, Lyderic Aubert, Sylvie Boa, Serge Ferracci, Robert Garnier

**Affiliations:** 1 French West Indies Toxicovigilance Coordination, Basse-Terre Hospital, Basse-Terre, France; 2 Research Unit EA 4651 Aliments Bioprocédés Toxicologie Environnements (ABTE), IFR146 ICORE, Normandie Université de Caen, Esplanade de la paix, Caen, France; 3 Department of Critical Care and Emergency Unit, Basse-Terre Hospital, Basse-Terre, France; 4 Santé publique France, French national public health agency, Regional unit (Cire) Antilles, Saint-Maurice, France; 5 Agence Régionale de Santé Guadeloupe, Monitoring and health alert unit, Gourbeyre, France; 6 Department of Critical Care and Emergency Unit, Pointe-à-Pitre University Hospital, Pointe-à-Pitre, France; 7 Poison and Toxicovigilance Centre, Fernand Widal Hospital, Paris, France

## Abstract

This retrospective case study analysed the incidence and symptoms of ciguatera fish poisoning (ciguatera) in Guadeloupe (French West Indies) between 2013 and 2016. Cases attending the emergency departments of the two public hospitals and the reports received by the regional health authority in charge of monitoring (ARS) were compiled. Two hundred and thirty-four cases of poisoning were observed, with a mean annual incidence of 1.47/10,000 (95% CI): 1.29–1.66), i.e 5 times higher than the previously reported incidence (1996–2006). The main species described as being responsible for poisoning were fish from the *Carangidae* family (n = 47) (jack), followed by fish from the *Lutjanidae* family (n = 27) (snapper), *Serranidae* family (n = 15) (grouper), S*phyraenidae* family (n = 12) (barracuda), and *Mullidae* family (n = 12) (goatfish). One case of lionfish ciguatera was observed. 93.9% of patients experienced gastrointestinal symptoms, 76.0% presented neurological signs (mainly paresthesia, dysesthesia and pruritus) and 40.3% presented cardiovascular symptoms (bradycardia and/or hypotension). A high frequency (61.4%) of hypothermia (body temperature <36.5 °C) was observed. This study reports for the first time the relatively high frequency of cardiac symptoms and low body temperature. The monitoring of ciguatera poisoning throughout the Caribbean region must be improved, notably after reef disturbance due to Irma and Maria major cyclones.

## Introduction

Ciguatera is the most common form of poisoning associated with fish consumption in the world, with about 10,000 to 50,000 cases per year^1^ or more^2^. Ciguatera is due to the consumption of fish containing ciguatoxins (CTXs), which are neurotoxins produced by microalgae of the *Gambierdiscus* and *Fukuyoa* genuses that are transferred through the food web. French overseas territories are particularly affected, with varying impacts from one territory to another throughout the world, but also locally from one archipelago to another. In the Pacific ocean, French Polynesia has a high annual incidence rate, estimated to be 18/10,000 in 2016^3^. In the Indian Ocean, the estimated annual incidence rate is lower: 0.2/10,000 between 2000 and 2010^4^. In the French West Indies (Lesser Antilles), the annual incidence was 0.3/10,000 in Guadeloupe and 0.2/10,000 in Martinique over the period 1996–2006^5^. However, much higher incidences have been observed over the same period in Caribbean countries adjacent to the French West Indies, such as Montserrat (58.6/10,000), Antigua (34.4/10,000) or the English Virgin Islands (19.9/10,000)^5^. This large intra-regional variability may reflect implementation of ciguatera detection and monitoring tools or may be due to the variable proliferation of ciguatera species from one territory to another. The epidemiology of ciguatera therefore remains difficult to assess and several authors have estimated that fewer than 20% of cases are reported^6,7^.

Clinically, during the acute phase of poisoning, the syndrome is predominantly characterized by gastrointestinal symptoms (nausea, vomiting, abdominal pain, diarrhea) and neurological symptoms (paresthesia, dysesthesia), but also muscular, cutaneous (pruritus) and cardiovascular symptoms (bradycardia, hypotension). Neurological symptoms may persist for several months. The symptoms may differ from one region of the world to another^8^. Predominantly neurological forms are observed in the Pacific. In the Indian Ocean, the syndrome is associated with neurological disorders, and hallucinations, loss of balance and depression have also been reported. Finally, in the Caribbean, gastrointestinal symptoms are often reported to be the predominant features during the early phase, followed by mainly neurological signs and symptoms, particularly involving the peripheral nervous system^9^. This variability from one region to another may be due to structural differences between CTX in each region, classified as P-CTX (Pacific-ciguatoxins), C-CTX (Caribbean-ciguatoxins) and I-CTX (Indian-ciguatoxins)^10^. Measures have been implemented in Guadeloupe to protect the population from ciguatera in the form of Prefectural orders (No. 2002–1249) listing the species that are prohibited for fishing and sale. This list is regularly updated as a result of progress in knowledge and according to the frequency of ciguatoxin contamination by the various species. Healthcare professionals are also required to notify potential cases of ciguatera to the Regional Health Authority (ARS). The ARS can carry out investigations, particularly on the remains of fish, in order to biologically confirm the presence of ciguatoxins in contaminated samples. A retrospective study was conducted to estimate the incidence and to analyze the symptoms and signs of ciguatera poisoning in Guadeloupe on the basis of data collected by the Guadeloupe ARS and data from cases of poisoning seen in the emergency departments of the 2 public hospitals of Guadeloupe (Pointe-à-Pitre University Hospital and Basse-Terre Hospital) between 2013 and 2016.

## Results

Over the 4-year study period, 113 cases were reported to hospital emergency departments and 123 cases were reported to the ARS, ie a total of 234 cases. Sixteen cases were excluded, 5 from Saint-Martin/Saint-Barthélémy and 11 duplicate cases derived from the two data sources. These 234 cases comprised 99 cases of isolated poisoning and 46 cases of collective poisoning (an average of 1.6 ± 1.5 people [range: 1 to 14] were poisoned during the same meal), i.e. a total of 145 cases of poisoning.

### Overall incidence and species involved

For an estimated population of 402,119 inhabitants in Guadeloupe, the overall annual incidence of ciguatera was 1.46/10.000 (95% CI: 1.27–1.65) over the study period. The number of cases was stable between 2013 and 2016, with 68, 45, 72 and 49 cases per year, respectively (non-significant regression line). Peaks were observed in November 2013 (n = 8), April and May 2015 (n = 7 and n = 9) and October-November 2016 (n = 7 and n = 6). On average, over the 4-year period, slightly more cases were observed between September and November (total number of cases over these 3 months: n = 49).

The incriminated species were mainly fish from the *Carangidae* family (n = 47) (jacks), followed by fish from the *Lutjanidae* family (n = 27) (snappers), *Serranidae* family (n = 15) (groupers), *Sphyraenidae* family (n = 12) (barracuda), and *Mullidae* family (n = 12) (goatfishes) (Table 1). Between 2013 and 2016, the number of cases of poisoning dropped sharply for great barracuda (from 8 cases in 2013 to 1 case in 2016) and grouper (7, 1, 4 and 4 cases each year from 2013 to 2016, respectively), while the number of cases due to jack fish increased during this period (11 cases in 2013, 5 cases in 2014, 15 cases in 2015 and 16 cases in 2016). The number of cases related to snapper and goatfish remained constant from year to year. Various fish species were incriminated, sometimes including prohibited species. Furthermore, three species: *Ocyurus chrysurus*, *Coryphaena hippurus* and *Centropomus undecimalis* not known to be responsible for CFP, were suspected to be involved in the present study (Table 1), but could possibly correspond to incorrect species identifications.

**Table 1:** Distribution of fish considered to be responsible for 145 cases of ciguatera poisoning (2 local fishes were suspected in 4 cases, and 3 local fishes were suspected in 1 case) according to hospital emergency department cases and regional monitoring network. Incriminated fish species are added, sometimes resulting in an overlap with species banned by Prefectural orders.

**Fish family** in *Latin* (and English)	**Local name of fish** in French (or English)**(***and corresponding species* **when name was specific)**	**n**	**Corresponding list of species known to be responsible for CFP** in *Latin* (and English)^**1**^
*Carangidae* (Jacks)	**Carangue**	47	*Caranx bartholomaei* (Yellow jack)^1,2^ *Caranx lugubris* (Black jack)^1,2^ *Caranx ruber* (Bar jack)^1,2^ *Caranx latus* (Horse Eye Jack)^1,2^ *Seriola dumerili* (Greater amberjack) *Seriola riviolana* (Almaco jack)
*Lutjanidae* (Snappers)	**Pagre, Sarde**	16	*Lutjanus jocu* (Dog Snapper)^1,2^ *Lutjanus apodus* (Schoolmaster Snapper)^1,2^
	**Vivaneau** (*Lutjanus buccanella*) (Black fin Snapper)^1,2^	10	
	**Colas** (*Ocyurus chrysurus*) (Yellowtail Snapper)*	1	
*Serranidae* (Groupers)	**Mérou, Grande gueule**	14	*Epinephelus morio* (Red grouper)^1^ *Mycteroperca* spp.^1^
	**Vieille à carreaux** (*Mycteroperca venenosa*) (Yellowfin grouper)^1,2^	1	
*Sphyraenidae* (Barracudas)	**Barracuda, grande bécune** (*Sphyraena barracuda*) (Great barracuda)^1,2^	12	
*Mullidae* (Goatfishes)	**Barbarin, Rouget Barbet** (*Mulloidichtys martinicus*)(Yellow goatfish)^1^	12	
*Muraenidae* (Morays)	**Murène, Congre** (*Gymnothorax funebris*) (Green Moray)^1,2^	4	
Coryphaenidae (Dolphins)	**Dorade** (*Coryphaena hippurus*) (Dolphin)*	3	
*Labridae* (Wrasses)	**Parroquette** (*Halichoeres radiatus*) (Pudding wife)^1^	3	
*Haemulidae* (Grunts)	**Gorette** ^**1**^	2	*Haemulon album* (Margate)^1^
*Priacanthidae* (Bigeyes)	**Poisson-soleil (***Priacanthus arenatus*) (Bigeye)^1^	1	
*Scombridae* (Tunas and mackerels)	**Thazard Cero** (*Scomberomorus regalis*) (Cero)^1^	1	
*Balistidae* (Triggerfishes)	**Bourse** (*Balistes vetula*) (Queen Triggerfish)^1^	1	
*Centropomidae*	**Brochet de mer** Snook (*Centropomus undecimalis*) (Snook)*	1	
*Scorpaenidae*	**Poisson-lion (***Pterois volitans*) (Lionfish)^1^	1	
Not described	Not described	21	—

^1^From Pottier *et al*.^9^.

^2^Species banned for sale and consumption: Prefectural orders (N°2002–1249) *These fish are not considered to be responsible for CFP at the present time.

### Human health effects of ciguatera

The clinical features of ciguatera fish poisoning were described for 196 of these 234 cases (92 emergency department cases and 104 ARS cases). A balanced proportion of men and women was observed (n = 88 and n = 84, respectively, sex not reported for 24 cases) and the mean age was 50.4 ± 17.1 years (range: 3 to 86 years), corresponding to a relatively older population (26% of patients were 60 years or older).

Clinically (Tables 2), 93.9% of patients experienced gastrointestinal symptoms such as nausea, vomiting, diarrhea or abdominal pain. Neurological signs were reported in 76.0% of patients with a predominance of peripheral nervous system symptoms (64.4%), paresthesia (42.4%), dysesthesia (35.6%) and pruritus (37.8%). Cardiovascular symptoms (bradycardia and/or hypotension) were also present in 40.3% of patients.

**Table 2:** Symptoms observed in cases of ciguatera poisoning in emergency department cases, regional monitoring network cases and cumulative data.

**Symptoms**	**Emergency department cases (n = 92)**	**Regional monitoring network (n = 104)**	**Cumulative data**
**Gastrointestinal**	97.8% (90/92)	90.4% (94/104)	93.9%
Diarrhea	82.6% (76/92)	77.9% (81/104)	80.1%
Vomiting	67.4% (62/92)	46.2% (48/104)	56.1%
Nausea	19.6% (18/92)	25.0% (26/104)	22.4%
Abdominal pain	37.0% (34/92)	58.7% (61/104)	48.5%
**Neurological**	65.2% (60/92)	85.6% (89/104)	76.0%
*Peripheral Nervous System*	50.0% (46/92)	80.0% (68/85)	64.4%
Paresthesia	34.8% (32/92)	50.6% (43/85)	42.4%
Dysesthesia	15.2% (14/92)	57.6% (49/85)	35.6%
Pruritus	18.5% (17/92)	54.8% (57/104)	37.8%
Myalgia	3.3% (3/92)	11.5% (12/104)	7.7%
Arthralgia	4.3% (4/92)	1.9% (2/104)	3.1%
*Central Nervous System*	27.2% (25/92)	18.3% (19/104)	22.4%
Vertigo/Dizziness/Loss of consciousness	21.7% (20/92)	16.3% (17/104)	18.9%
Visual disturbance	3.3% (3/92)	0.0% (0/104)	1.5%
Headache	6.5% (6/92)	7.7% (8/104)	7.1%
Hallucinations	0.0% (0/92)	1.0% (1/104)	0.5%
**Cardiovascular (hypotension or bradycardia)**	73.9% (68/92)	10.6% (11/104)	40.3%
Hypotension	33.7% (31/92)	8.7% (9/104)	20.4%
Bradycardia	70.7% (65/92)	4.8% (5/104)	35.7%
*Other cardiovascular signs*			
Hypertension	8.7% (8/92)	0.0% (0/104)	4.1%
Tachycardia	6.5% (6/92)	1.9% (2/104)	4.1%
**Others**			
Hypothermia (T°C <36.5)	61.4% (51/83)	NR*	
Asthenia/Fatigue	33.7% (31/92)	42.3% (44/104)	38.3%

*Hypothermia was not investigated in regional monitoring network cases.

**Figure 1:**
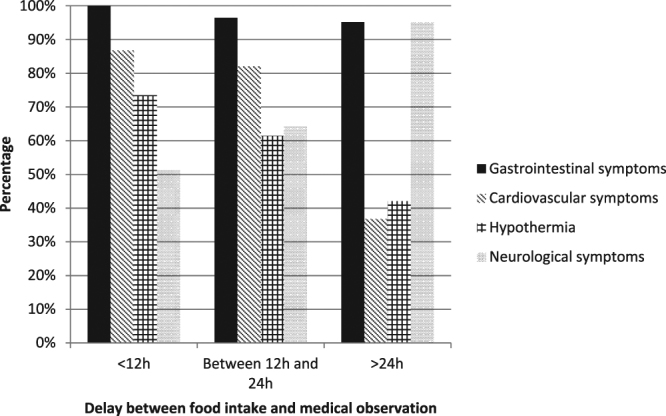
Symptoms observed at the initial medical examination in hospital emergency departments according to the time between food intake and medical examination: less than 12 hours (n = 37), between 12 and 24 hours (n = 28) and more than 24 hours (n = 21). Cardiac symptoms were observed in 86.8% of cases when the patient was seen in less than 12 hours, in 82.1% of cases when the patient was seen between 12 and 24 hours and in 36.8% of cases when the patient was seen more than 24 hours after food intake. The frequency of hypothermia decreased with observation time (73.5%, 62% and 42%, respectively). Neurological symptoms were more often reported when the patient attended the emergency department more than 24 hours after food intake (95.2% of cases).

Significant differences in terms of symptoms and signs were observed between the two data sources (hospital emergency departments and private physicians/ARS reports). Cardiovascular symptoms were predominant in emergency department cases, as 70.7% of cases presented bradycardia (heart rate ≤ 60 bpm) and 33.7% presented hypotension (systolic blood pressure < 90 mmHg). Cardiovascular symptoms were essentially observed when the patient attended the emergency department within 12 hours after eating ciguatera fish (87.8%). Nevertheless, cardiovascular symptoms were also observed in one-third of patients who attended the emergency department more than 24 hours after their toxic meal (Fig. 1). Severe bradycardia (heart rate ≤ 40 bpm) was observed in 16.3% of patients (n = 15), and atropine was used in 14 patients. Hypothermia (body temperature <36.5 °C) was reported in 61.4% of patients and hypothermia was correlated with bradycardia (correlation coefficient: 0.40, p < 0.001) and hypotension (correlation coefficient: 0.44, p < 0.001): lower blood pressure or pulse was associated with lower body temperature. The ARS cases most often presented neurological symptoms (85.6% of cases), while cardiac symptoms were reported less frequently (10.6% of cases).

## Discussion

Monitoring of ciguatera is complex and usually requires the creation of dedicated monitoring networks, collecting cases reported by healthcare professionals or individuals. Monitoring is therefore based on voluntary notifications and the ability of each network to encourage healthcare professionals to notify these cases. Moreover, the diagnosis of ciguatera is not always easy, as the symptoms are nonspecific and only laboratory analysis of the remains of the fish consumed can confirm ciguatera contamination of the fish. The present study was based on cases managed by the emergency departments of the two public hospitals in Guadeloupe and notifications to the competent authority in charge of monitoring (namely the Guadeloupe ARS). The mean annual incidence of ciguatera observed in this study was 1.46/10,000 pa, an incidence almost fivefold higher (RR: 4.87, 95% CI: 3.29–7.21) than that reported for the period 1996–2006 (0.3/10,000)^7^. Nevertheless, this estimate for the period 1996–2006 was based solely on reports received by health authorities, whereas our study collated these cases with all cases observed in emergency departments. If only the cases reported to health authorities (n = 123) were taken into account, this annual incidence would be only 0.76/10,000, twice that of the previous period. Emergency departments must therefore be encouraged to report cases of ciguatera, as only 11 of the 111 cases seen in emergency departments were notified to ARS. The number of cases remained fairly stable from year to year during the study period, despite bans on the sale of species potentially responsible for ciguatera and awareness campaigns targeting fishermen and the general population. Nevertheless, the number of cases of ciguatera related to barracuda and grouper decreased, which could be a direct result of these information campaigns. *Carangidae* fish were the species most frequently responsible for ciguatera. Cases of ciguatera were also observed after eating fish not previously known to contain ciguatoxins, as a case of ciguatera was observed in a patient who had consumed lion fish (*Pterois volitans*). Clinical signs were typical of ciguatera fish poisoning, including paresthesia, loss of consciousness, gastrointestinal signs (vomiting, diarrhea), impaired visual acuity and bradycardia/hypotension that resolved over 24 hours. In recent years, lionfish has become an invasive species in the West Indies^11^ and lionfish consumption is increasingly widespread. Lionfish is considered to be responsible for ciguatera in Saint Martin, but is not prohibited for sale and consumption in Guadeloupe, as testing of this species did not reveal the presence of ciguatoxins until at least 2015 in Guadeloupe, in contrast with Saint-Barthélemy^12^. In the future, it will therefore be necessary to analyze potential cases of ciguatera related to lionfish consumption, by performing ciguatoxin assays on consumed fish remains. Lionfish is also heavily contaminated by chlordecone pesticide residues, and should therefore also be banned for consumption for this reason^13^.

The present study showed that cardiac symptoms were very common in Guadeloupe (40.3% of patients), whereas the last review of the literature on ciguatera reported only few cardiac symptoms in the Caribbean region^7^. Only one of the six studies reported cardiovascular symptoms, with 3% of bradycardia and 33% of arrhytmia^14^. The high frequency observed in our study can be partly attributed to the case-finding methodology used in this study, as one-half of cases were derived from hospital emergency departments and patients attending emergency departments probably correspond to the most severe cases. Bradycardia could also constitute a discriminating factor for the diagnosis of ciguatera poisoning by emergency physicians, as, although cardiovascular symptoms have been poorly described in the literature in the Caribbean, the emergency physicians interviewed in the context of this study reported that they considered a possible diagnosis of ciguatera in patients presenting with bradycardia following a fish meal. Finally, the discordant results between our study and other studies conducted in the Caribbean could be related to a more general observation bias of all ciguatera studies: since cardiovascular signs are observed during the early hours of poisoning, their frequency will depend on the modalities of recruitment in each case series. Such discordant results have already been observed in Polynesia: Gatti *et al*.^15^ reported bradycardia in 75% of cases and hypotension in 43% of cases in a hospital cohort series of 124 patients in Tahiti (French Polynesia) between 1999 and 2005, while Chateau-Degat *et al*.^16^ reported no cases of bradycardia among the 800 reports received by the Polynesian monitoring network for the years 2007–2008. In fact, cardiac symptoms were first described in the Caribbean by Lawrence *et al*.^17^, (1980) but were reported to resolve within 12 hours. In the present study, the high frequency of cardiac symptoms, their severity (bradycardia/hypotension) and their persistence after 24 hour for 33.3% of patients, suggest that cardiovascular signs could be an integral part of the clinical features of ciguatera observed in the West Indies, but further studies are required to confirm this hypothesis.

Finally, a high incidence of hypothermia (61.4%) was observed during the acute phase of poisoning in cases treated in hospital emergency departments. Hypothermia was first described in 2009 in the Polynesian hospital cohort reported by Gatti *et al*.^15^, in which hypothermia was observed in 60% of patients and then in one-third of cases of a ciguatera epidemic in Germany in 2012^18^. Hypothermia has also been observed in several animal models (chick, mouse)^19–21^. In the present study, hypothermia was correlated with bradycardia and hypotension, but we cannot conclude that hypothermia is secondary to cardiovascular effects due to a defense mechanism, or that hypothermia is directly due to a specific biotoxin. Indeed, the cardiovascular effects of ciguatoxins are mediated by binding of ciguatoxins to voltage-gated sodium channels, which remain open. However, other substances binding to voltage-gated sodium channels (such as pyrethroid pesticides or scorpionic toxins) are not known to cause hypothermia. Ciguatoxins may also have a direct action on the central nervous system^21^ by acting on thermoregulation centers in the pre- and supra-optic areas of the hypothalamus. Other biotoxins may be involved, including maitotoxins, also synthesized by *Gambierdiscus*^22^ and localized in the viscera of contaminated fish, or brevetoxin, both of which having shown a hypothermic effect in mice and rats^23^. Further research could therefore be performed to determine whether hypothermia should be considered to be a diagnostic feature of ciguatera.

The presence of *Gambierdiscus* and *Fukuyoa microalgae* in the Caribbean region has also been well documented^24,25^ and the exact species and the corresponding toxinic pattern of *Gambierdiscus* algae present in Guadeloupe sea water and their prevalence from a public heath viewpoint also need to be determined.

This study was not sufficiently powerful to distinguish seasonal variations, especially seasonal variations of water temperature that have been described to be correlated with an increased incidence of ciguatera^4^. We simply observed that slightly more cases were observed during the months of September, October and November, corresponding to the period with the warmest sea temperatures in the West Indies. Moreover, the retrospective nature of this study did not allow analysis of the kinetics of resolution of the symptoms, especially cardiovascular and neurological symptoms, which would have required follow-up of patients over time. At last, common regional names of fish were generally reported and species were not formally identified by a specialist. Despite the creation of an ARS monitoring network, few notifications have been received (only 10% of the cases observed in emergency departments were reported to the regional monitoring network). From scientific and public health perspectives, there is a need to develop this regional monitoring network in Guadeloupe and in all of the Caribbean region by developing a standardized CFP survey. This is particularly important since the major hurricanes Irma and Maria have disturbed the reefs in 2017, major cyclones known to be a strong predictor of ciguatera poisoning^26^.

## Conclusion

A study of ciguatera poisoning based on both reports received by the monitoring network and cases managed in hospital emergency departments allowed more reliable evaluation of the incidence and characteristics of ciguatera in the Guadeloupe archipelago. The methodology used resulted in a mean annual incidence of 1.47/10,000, which was fivefold higher than the previously reported incidence. This incidence is not declining despite the protective measures set up by the authorities over recent years. A prospective case study, guided by a standardized survey, would allow a more accurate description of the clinical features and the course of ciguatera poisoning in the French West Indies. In particular, such a study should specify the frequency of hypothermia and cardiovascular symptoms, which are probably underestimated in published series.

## Material and Methods

All cases of ciguatera reported to the ARS between 1 January 2013 and 31 December 2016 were analyzed, except for those from Saint-Martin and Saint-Barthélemy. Each report contains an individual survey with the date, category of the notifier (private physician, hospital physician or general public), the number of people poisoned, the fish incriminated and the clinical signs, defined according to the following items: “diarrhea”, “asthenia”, “dysesthesia”, “paresthesia”, “abdominal pain”, “vomiting”, “nausea”, “itching”, “malaise”, “fever”, “vertigo” or a free field concerning the other clinical signs observed. In parallel, a search for ciguatera cases was carried out during the same period in the emergency department databases of the two public hospitals using key words (“cigua”, “fish”) or ICD-10 code T61.0: “Ciguatera fish poisoning”. Each case was assessed by an experienced toxicologist and cases corresponding to ciguatera poisoning were included in the study. The case selection criteria were consumption of fish followed,, within 24 hours, by gastrointestinal signs (diarrhea, vomiting, nausea, abdominal pain), neurological signs (paresthesia, dysesthesia, pruritus) or cardiac signs (bradycardia and/or hypotension). The following characteristics were recorded for each case: age, sex, medical history, date of poisoning and date of management, incriminated species, number of people poisoned, clinical features (classified as gastrointestinal signs, neurological signs, cardiac signs and other signs) and treatment. When several measurements of heart rate, blood pressure or body temperature were available, the lowest measurement before treatment was recorded. Duplicate cases between ARS reports derived from hospital physicians and cases extracted from hospital emergency department databases were deleted on the basis of date, sex, age and the type of fish incriminated. All cases were taken into account (ARS cases and emergency department cases) to assess the overall incidence of ciguatera and the species involved. Only cases with a complete emergency report or an individual ARS declaration form (indicating the clinical signs) were used to evaluate the clinical characteristics of poisoning.

Quantitative variables were expressed as mean ± standard deviation, and qualitative variables were expressed as percentage and relative risk, together with the 95% confidence interval. Spearman’s correlation coefficient was used to analyze associations between heart rate, blood pressure and body temperature. A limit of p < 0.05 was considered to be significant. All analyses were performed with R v.3.0.2 software.

All methods were carried out in accordance with STROBE recommendations.

The CIRE and the two hospitals determined it to be in accordance with the French regulations (CNIL authorization obtained) and that the criteria for waived patient authorization had been met. All cases of ciguatera must be reported to ARS in Guadeloupe for investigations. The database created for this study has been anonymised.

### Data availability statement

The datasets generated during and/or analysed during the current study are available from the corresponding author on reasonable request.
